# Potential immunosuppressive effects of *Escherichia coli* O157:H7 experimental infection on the bovine host

**DOI:** 10.1186/s12864-016-3374-y

**Published:** 2016-12-21

**Authors:** E. Kieckens, J. Rybarczyk, R. W. Li, D. Vanrompay, E. Cox

**Affiliations:** 1 Laboratory of Immunology, Faculty of Veterinary Medicine, Ghent University, Salisburylaan 133, 9820 Merelbeke, Belgium; 2 USDA-ARS, Bovine Functional Genomics Laboratory, Beltsville, MD USA; 3 Laboratory of Immunology and Animal Biotechnology, Faculty of Bioscience Engineering, Ghent University, Coupure Links 653, 9000 Ghent, Belgium

**Keywords:** RNA-Seq, *Escherichia coli* O157:H7, Cattle, Immunosuppression

## Abstract

**Background:**

Enterohaemorrhagic *Escherichia coli* (EHEC), like *E. coli* O157:H7 are frequently detected in bovine faecal samples at slaughter. Cattle do not show clinical symptoms upon infection, but for humans the consequences after consuming contaminated beef can be severe. The immune response against EHEC in cattle cannot always clear the infection as persistent colonization and shedding in infected animals over a period of months often occurs. In previous infection trials, we observed a primary immune response after infection which was unable to protect cattle from re-infection. These results may reflect a suppression of certain immune pathways, making cattle more prone to persistent colonization after re-infection. To test this, RNA-Seq was used for transcriptome analysis of recto-anal junction tissue and ileal Peyer’s patches in nine Holstein-Friesian calves in response to a primary and secondary *Escherichia coli* O157:H7 infection with the Shiga toxin (Stx) negative NCTC12900 strain. Non-infected calves served as controls.

**Results:**

In tissue of the recto-anal junction, only 15 genes were found to be significantly affected by a first infection compared to 1159 genes in the ileal Peyer’s patches. Whereas, re-infection significantly changed the expression of 10 and 17 genes in the recto-anal junction tissue and the Peyer’s patches, respectively. A significant downregulation of 69 immunostimulatory genes and a significant upregulation of seven immune suppressing genes was observed.

**Conclusions:**

Although the recto-anal junction is a major site of colonization, this area does not seem to be modulated upon infection to the same extent as ileal Peyer’s patches as the changes in gene expression were remarkably higher in the ileal Peyer’s patches than in the recto-anal junction during a primary but not a secondary infection. We can conclude that the main effect on the transcriptome was immunosuppression by *E. coli* O157:H7 (Stx^−^) due to an upregulation of immune suppressive effects (7/12 genes) or a downregulation of immunostimulatory effects (69/94 genes) in the ileal Peyer’s patches. These data might indicate that a primary infection promotes a re-infection with EHEC by suppressing the immune function.

## Background

Enterohaemorrhagic *Escherichia coli* (EHEC), such as *E. coli* O157:H7, are frequently detected in faecal cattle samples at slaughter (6.3% in Belgium, *n* = 1281) [1]. Cattle are the main natural reservoir, do not show clinical signs upon infection and can remain asymptomatic carriers for a very long period. If humans become infected by consuming contaminated food, mainly inadequately cooked beef products, the consequences can be severe [2]. After ingestion and subsequent colonization of the human colon, EHEC releases Shiga toxins causing microvascular endothelial injuries, which might lead to bloody or non-bloody diarrhea, haemorrhagic colitis and the haemolytic uremic syndrome [3, 4].

Recently, the prevalence of EHEC was studied in 12 Belgian cattle herds of which some animals were diagnosed as EHEC-positive at slaughter [5]. Longitudinal follow up of herds showed that faecal samples were intermittent positive, while some animals were suggested to have a chronic excretion over a period of at least 6–12 weeks was observed. Of the intermittent shedders, one animal was positive at the beginning of the study and was also excreting when sampled 11 months later. Furthermore, the shedding patterns showed that positive animals can shed different strains at different sample points [6]. Other studies showed that positive animals became culture negative within 2–3 months after the first testing [7]. The immune response of the animals against the EHEC strains, which has been investigated in few studies, might explain these excretion patterns. One study demonstrated that antibodies against the O157 lipopolysaccharide (LPS) and Shiga toxin-1 and -2 (Stx1, Stx2) frequently occur in bovine sera and colostrum upon experimental infection [8], but this response could not clear the infection, as infected animals secreted bacteria over a period of months [4]. Another study on two farms demonstrated that faecal excretion was not always correlated with *E. coli* secreted protein A (EspA), intimin and, translocated intimin receptor (Tir) specific serum antibody responses. In contrast, 87.5% of the animals showed a serum antibody response against *Escherichia coli* secreted protein B (EspB) at the same time that their faecal sample was positive for EHEC O157, O26 or, O111 or 6 weeks after a positive faecal sample. These antibodies persisted, even when shedding had ceased, until the animals were slaughtered, which was 2–8 months later, whereas EspA-specific antibodies disappeared within 2 months [9]. These results indicate that farm animals, which develop an immune response after infection, can become reinfected by different EHEC strains as evidenced by intermittent excretion which may reflect suppression of certain pathways of the immune system by the primary infection, making cattle more prone to persistent colonization by a subsequent infection.

In 2003, Naylor et al. [10] described the preference of *E. coli* O157:H7 for the terminal rectum up to the recto-anal junction (RAJ) as primary site for colonization. This site is characterized by a high density of lymphoid follicles. Predilection for epithelium above mucosa-associated tissue could be important for modulating the immune system. Indeed, EHEC O157 is capable of suppressing cell-mediated immune responses in cattle by targeting lymphocytes via their Shiga toxins [8, 11], but enterocytes do not have receptors for these toxins, suggesting that close contact with the immune system might be necessary. Here, the ileal Peyer’s patches might play a role as they are of major importance for the mucosal immune responses in cattle [12]. In this study, a Stx negative strain was used for biosafety reasons. Nevertheless immunomodulating effects of other virulence factors of *E. coli* O157:H7 have been described. The H7 flagellin, bacterial LPS and type IV pilus have been shown to induce proinflammatory responses upon EHEC infection. On the contrary, it has been observed that EHEC as wells as EPEC strains could suppress NF-κB and MAPK activation as well as IκB degradation, [13] and could inhibit the production of proinflammatory cytokines IL-8 and IL-6, early in the infection by different LEE- and non-LEE encoded effectors (Tir, NleB, NleC, NleD, NleE, NleH1 and NleH2) [14]. Clearance of EHEC O157 is associated with an up-regulation of Th-1 associated transcripts within the rectal mucosa, the principle site of colonization [10, 15], suggesting that a cellular component of the adaptive immune response may be important in EHEC O157 control.

In this study we wanted to obtain insights in genes involved in an immunosuppressive effect of an *E. coli* O157:H7 Stx negative strain. In our experimental infections prolonged excretion was observed after a second infection with this strain. Transcriptome analysis of the ileal Peyer’s patches and the RAJ from calves was performed using RNA-seq technology. Samples were taken from animals infected either once or twice which have never been in contact with *E. coli* O157:H7.

## Methods

### Bacterial strain

The *E. coli* O157:H7 strain NCTC 12900, a well-characterized Shiga-toxin negative *E. coli* O157:H7 strain of human origin with naladixic acid resistance [16] was used for experimental infections in calves. We used this Stx-negative strain for biosafety reasons. Bacteria were grown overnight in Luria Bertani broth (LB) with aeration (200 rpm) at 37 °C, harvested by centrifugation (11 337 × g, 5 min), re-suspended in sterile phosphate-buffered saline (PBS) to a concentration of 10^10^ CFU/10 ml and subsequently used for experimental infections.

### Experimental infection of calves and sample collection

**Fig. 1:**
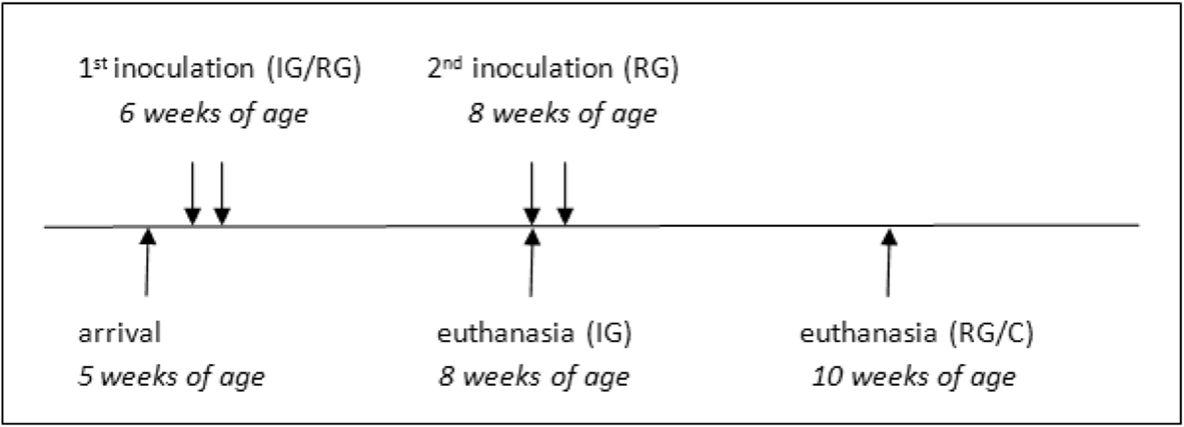
Experimental setup

Nine 5-week-old Holstein-Friesian calves were randomly assigned to three groups (primary infection, re-infection and uninfected control; *n* =3), each reared in separate boxes in isolation units (Fig. 1). These animals were screened to be seronegative for intimin, EspA and EspB, as well negative for faecal shedding of *E. coli* O157:H7 and non-O157:H7. The animals were milk-fed from their arrival until the end of the experiment and allowed free access to hay, water and grain-based pellets. The milk-uptake gradually decreased as the animals were able to digest more pellets, to allow a normal development of the gastro-intestinal tract.

Six animals were inoculated at the age of 6 weeks with 10^10^ CFU during two consecutive days as previously described by Kieckens et al. [17]. Two weeks after infection, three animals were euthanized (Infection Group, IG). At the same time, the remaining three infected animals were re-infected and euthanized 2 weeks later at 10 weeks of age (Re-infection Group, RG). At that time, the three uninfected control animals (Control Group, C) of the same breed and age, were also euthanized (Fig. 1). Euthanasia was carried out by captive bolt and samples from the ileal Peyers’ patches and the RAJ were collected for RNA isolation to analyze the transcriptome profiles by RNA-Seq. Hereto, tissue samples of 1 cm^2^ were cut and rinsed in sterile cold PBS (4 °C) and immediately frozen in liquid nitrogen and stored at −80 °C until RNA purification. All experimental and animal management procedures were undertaken in accordance to the requirements of the animal care and ethics committee of the Faculty of Veterinary Medicine, Ghent University, Belgium (EC2011/082).

### Excretion of *E. coli* O157:H7

Faecal samples were analyzed immediately after sampling, as described by Vande Walle et al. [18]. Briefly, ten gram of faeces was homogenized in 90 ml sterile modified tryptone soy broth (Oxoid Ltd., Hanst, United Kingdom) supplemented with 20 mg/liter novobiocin (Sigma Aldrich, St. Louis, MO, USA). Enumeration of *E. coli* O157 was performed by plating 10-fold serial dilutions onto MacConkey agar supplemented with sorbitol, cefixime, tellurite and nalidixic acid (NalCT-SMAC) (Merck, Darmstadt, Germany) and incubating the plates at 37 °C for 18 h. The remaining broth was enriched for 6 h at 42 °C and subjected to immunomagnetic separation (IMS) using Dynabeads (Invitrogen, Merelbeke, Belgium), according to the manufacturer’s instructions. Finally, 100 μl was plated onto NalCT-SMAC agar and incubated for 18 h at 37 °C. Selected sorbitol-negative colonies were confirmed by the O157-specific latex agglutination assay (Oxoid Ltd., Basingstoke, United Kingdom).

### RNA extraction and sequencing using RNA-seq

Total RNA was extracted from the tissues using the Qiagen RNeasy mini kit as described by the manufacturer. Briefly, the samples were ground to a fine powder under liquid nitrogen using a mortar and pestle and homogenized. Then, the lysate was further processed as instructed by Qiagen. The RNA purity was verified using NanoDrop Technology (Thermo Fisher Scientific, USA) and the RNA concentration was measured. High-quality RNA (260/280 nm ratio ~ 2.0; RNA Rin# > 8.0) was processed using an Illumina TruSeq RNA sample prep kit following manufacturer’s instruction (Illumina, San Diego, CA, USA). Individual RNA-Seq libraries were pooled based on their respective sample-specific 6-bp adaptors and sequenced at 50 bp/sequence read using an Illumina HiSeq2000 sequencer.

### Data analysis and bioinformatics

The mean number of raw reads generated per sample in the study was 18,910,480.56 ± 5,610,677.59 (mean ± SD; *n* =18). SolexaQA was used for trimming and filtering using default parameters. The resultant reads with < 40 bp in length were discarded. After performing trimming and filtering, the final number of reads for the genome alignment was 16,042,336.72 ± 5,046,779.84 (mean ± SD).

The resultant quality reads were aligned to the bovine reference genome (UMD 3.1) using TopHat2 (v2.0.6) [19] using the following parameters: mismatches allowed: 2 bp; and max insertions: 3 bp and max deletions: 3 bp (using bowtie2 v2.0.2 with INDEL allowed). The SAM output files from the TopHat alignment, along with the GTF file from ENSEMBL bovine genebuild v67.0, were used in the Cuffdiff program in the Cufflink package (v2.0.2) to test for differential gene expression. Mapped reads were normalized based on the upper-quartile normalization method. Cuffdiff models the variance in fragment counts across replicates using the negative binomial distribution [20].

Results were considered significant for *p* <0.05. Differentially expressed genes identified in the transcriptome were further analyzed using GeneOntology (GO) analysis (https://github.com/tanghaibao/goatools) after FDR correction for FDR <0.1. A Fisher ‘s exact test was used for enrichment of certain GO terms. A multiple correction control (permutation to control false discovery rate, FDR) was implemented to set up the threshold to obtain the list of significantly over-represented GO-terms as previously described [21].

IPA (Ingenuity Pathway Analysis) and Path designer was used to visualize connections between differentially expressed genes.

The fold change was reported in the result section for every gene in brackets.

## Results

### Excretion of *E. coli* O157:H7 (Stx^−^)

**Fig. 2:**
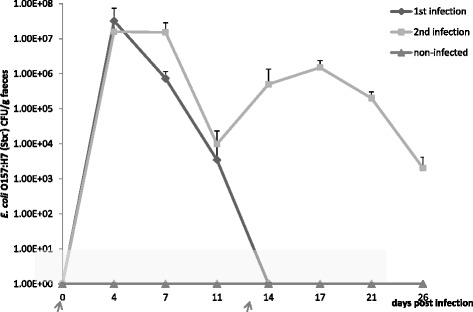
Average faecal excretion of infected animals. Error bars indicate standard deviations. Arrows on the x-axis indicate the timepoint of first and second infection

Average excretion patterns of the infected animals are shown in (Fig. 2).Animals were negative before infection and the highest peak of bacteria in faeces was detected at day 4 post infection. Within 14 days after the primary infection, all animals of the primary infection group became negative. The second infection gave rise to a lower level of bacterial shedding but these animals were still shedding bacteria at euthanasia.

### Transcriptome analysis of gut tissues

A total of 21,046 genes were detected at least once in one of the nine RAJ samples or in one of the nine ileal Peyer’s patches samples. The number of genes expressed per sample was 18,753.22 ± 276.94 (Mean ± SD). The number of genes with mean hits ≥ 5.0 was 16,788 whereas the core transcriptome of the RAJ and the ileal Peyer’s patches consisted of 16,948 and 17,197 genes, respectively. This core transcriptome incorporates genes represented by at least one sequence hit in each of the samples tested.

We used the transcript abundance classification as described by Li et al. [21]. Assuming 300,000 mRNA molecules per cell, approximately 70.68% of genes transcribed in the RAJ and 70.55% of genes transcribed in the ileal Peyer’s patches can be classified into “very rare” with a relative abundance of ≤15 molecules per cell (Table 1), followed by “rare” (16–99 molecules per cell) at 16.18 and 17.29%, respectively. “Not expressed” genes (0 molecules per cell) were calculated as 11.17 and 10.18%, whereas “moderately abundant” genes (100–500 molecules per cell) accounted for 1.74 and 1.78%. “Abundant” genes were only for 0.23 and 0.21% part of the transcriptome.

**Table 1:** Transcript abundance in the recto-anal junction and Ileal Peyer’s patches

Transcript category	Recto-anal junction	Ileal Peyer’s patches
Not expressed	11.17%	10.18%
Very rare	70.68%	70.55%
Rare	16.18%	17.29%
Moderately abundant	1.74%	1.78%
Abundant	0.23%	0.21%

### Genes significantly influenced by *E. coli* O157:H7 experimental infections

**Fig. 3:**
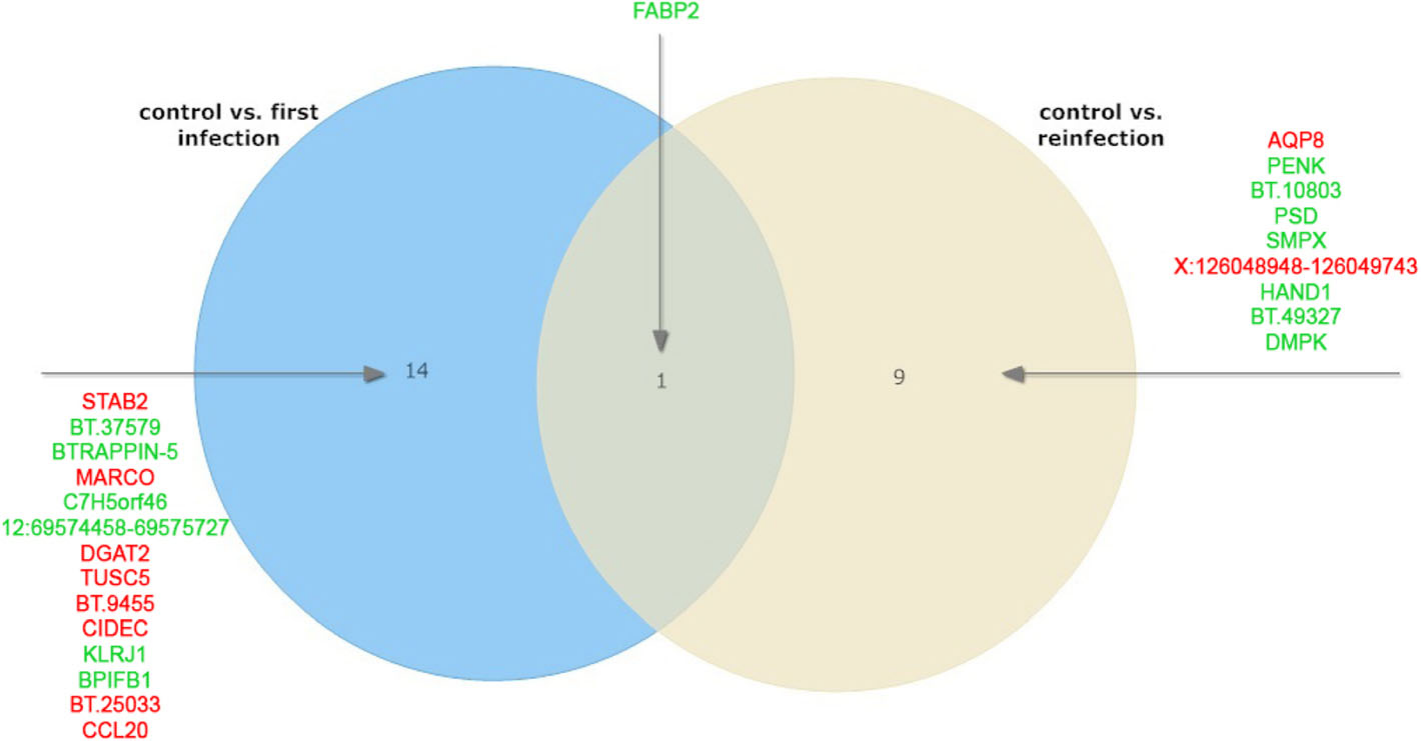
Venn diagram of differentially expressed genes in the recto-anal junction

**Fig. 4:**
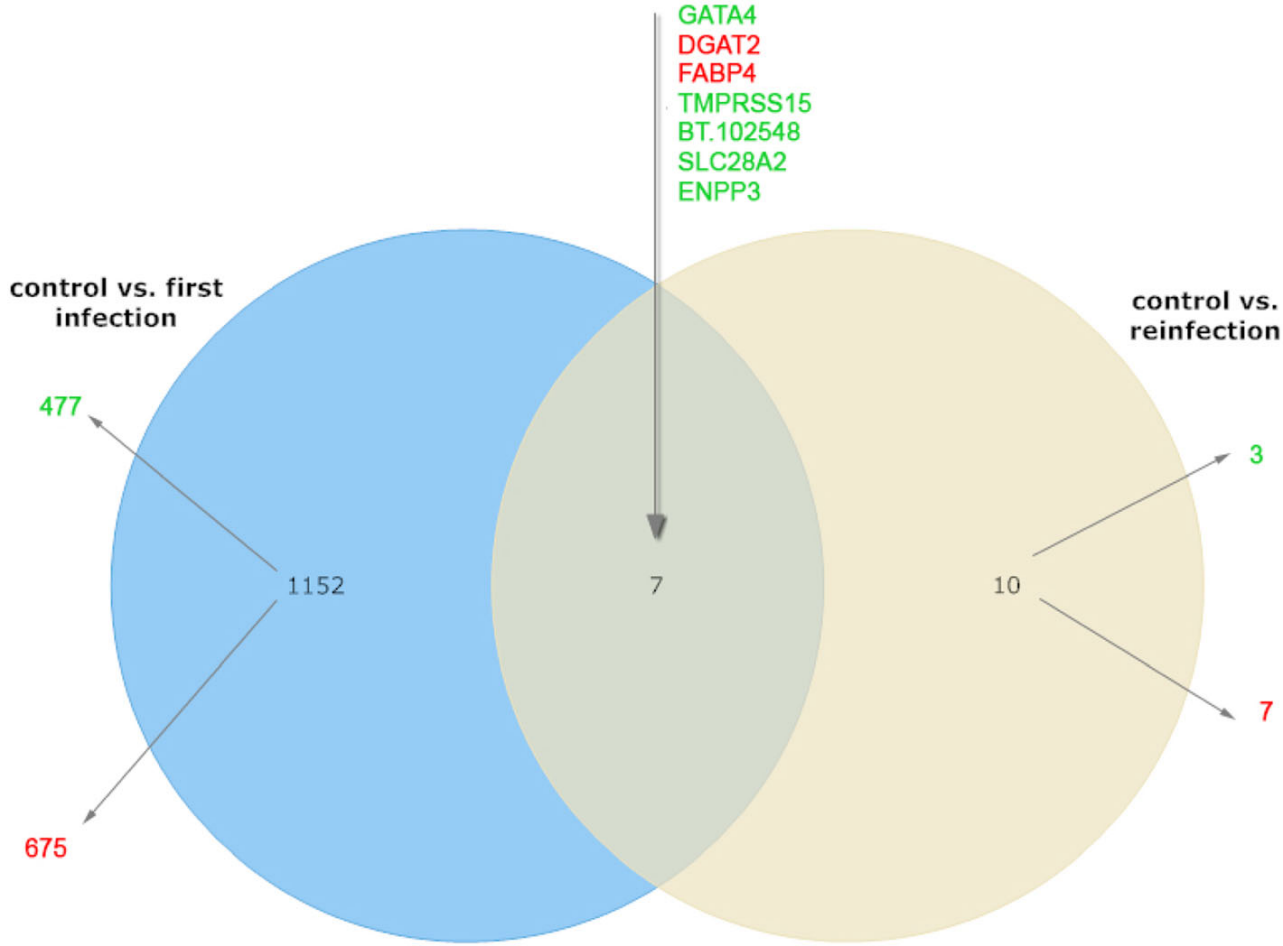
Venn diagram of differentially expressed genes in ileal Peyer’s patches

In the RAJ, the primary site of *E. coli* O157:H7 colonization in cattle, fifteen genes were found to be significantly affected by a primary infection with *E. coli* O157:H7 whereas ten genes were affected after re-infection compared to the uninfected control group (false discovery rate FDR < 0.1). Only one gene (*FABP2*) appeared to be significantly impacted by both primary infection and re-infection with *E. coli* O157:H7 (Fig. 3). Three out of fifteen genes that were significantly affected during the primary infection could be linked to an immune function (*KLRJ1, MARCO, CCL20*); one was upregulated and two downregulated (Table 2). In the ileal Peyer’s patches 1159 genes were significantly influenced by a primary infection compared to the control group and only seventeen genes were significantly affected by the re-infection compared to the same control, indicating a larger effect on the transcriptome during primary infection compared to a re-infection. Seven genes were significantly different regulated after a primary as well as after a re-infection (Fig. 4). The function of 103 out of 1159 genes that were differently regulated during the primary infection could be traced back to the immune system.

**Table 2:** Overview of numbers of up- and downregulated genes in respect to their effect on the immune system

Immune stimulating effect			Immune suppressive effect	
	# upregulated	# downregulated	# upregulated	# downregulated
Recto-anal junction	/	2	1	/
Ileum + PP	25	67	6	5

### Gene ontology (GO) analysis

A total of 15 genes were significantly affected by a primary infection with EHEC in the RAJ at a cutoff FDR <10%. Enrichment with Bonferroni corrected *P* value = 0.0115 reveals three genes possessing scavenger receptor activity (GO:0005044) and three genes having cargo receptor activity (GO:0038024). In the Peyer’s patches ileum, 1159 genes were significantly affected by a primary EHEC infection. After enrichment, we could identify 997 genes linked to a molecular function (GO:0003674), 882 genes to biological processes (GO:0008150), 673 genes were linked to a cellular process (GO:0009987) and 561 genes to a single-organism process (GO:0044699). Also GO-terms for metabolic process (GO:0008152), single-organism cellular process (GO:0044763), catalytic activity (GO:0003824), response to stimulus (GO:0050896), cellular response to stimulus (GO:0051716), nitrogen compound metabolic process (GO:0006807), single-organism metabolic process (GO:0044710), hydrolase activity (GO:0016787), small molecule metabolic process (GO:0044281), and response to stress (GO:0006950) were found for 492, 488, 405, 329, 247, 230, 223, 204, 145 and 123 genes, respectively, out of 1159 genes that were significantly impacted. GO-terms for <100 genes were not reported in this manuscript.

### Regulatory gene networks

**Fig. 5:**
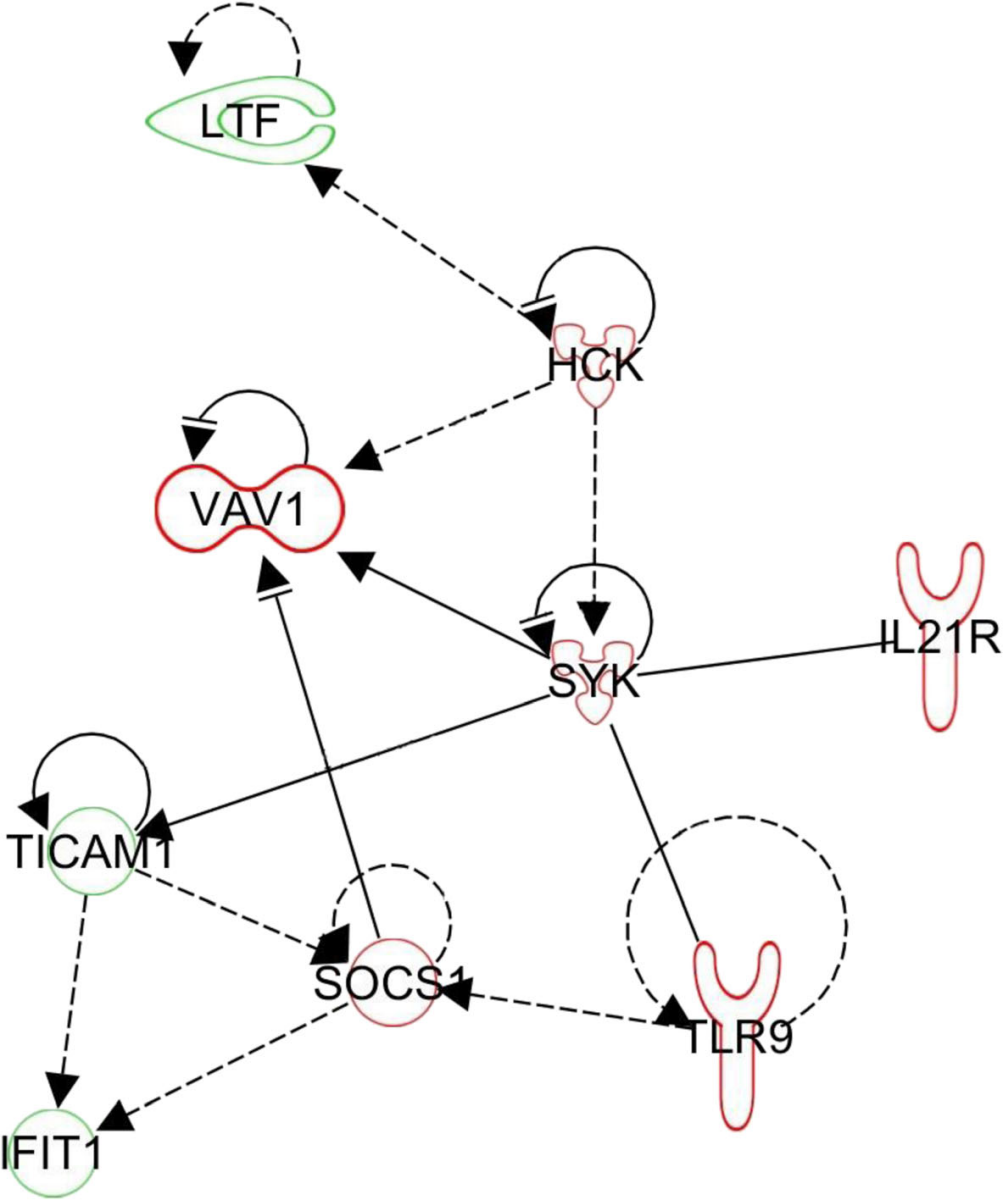
Regulatory network related to antimicrobial response impacted in the ileal Peyer’s patches of calves after a primary infection with *E. coli* O157:H7. Up- and downregulation is represented by green and red colours, respectively

**Fig. 6:**
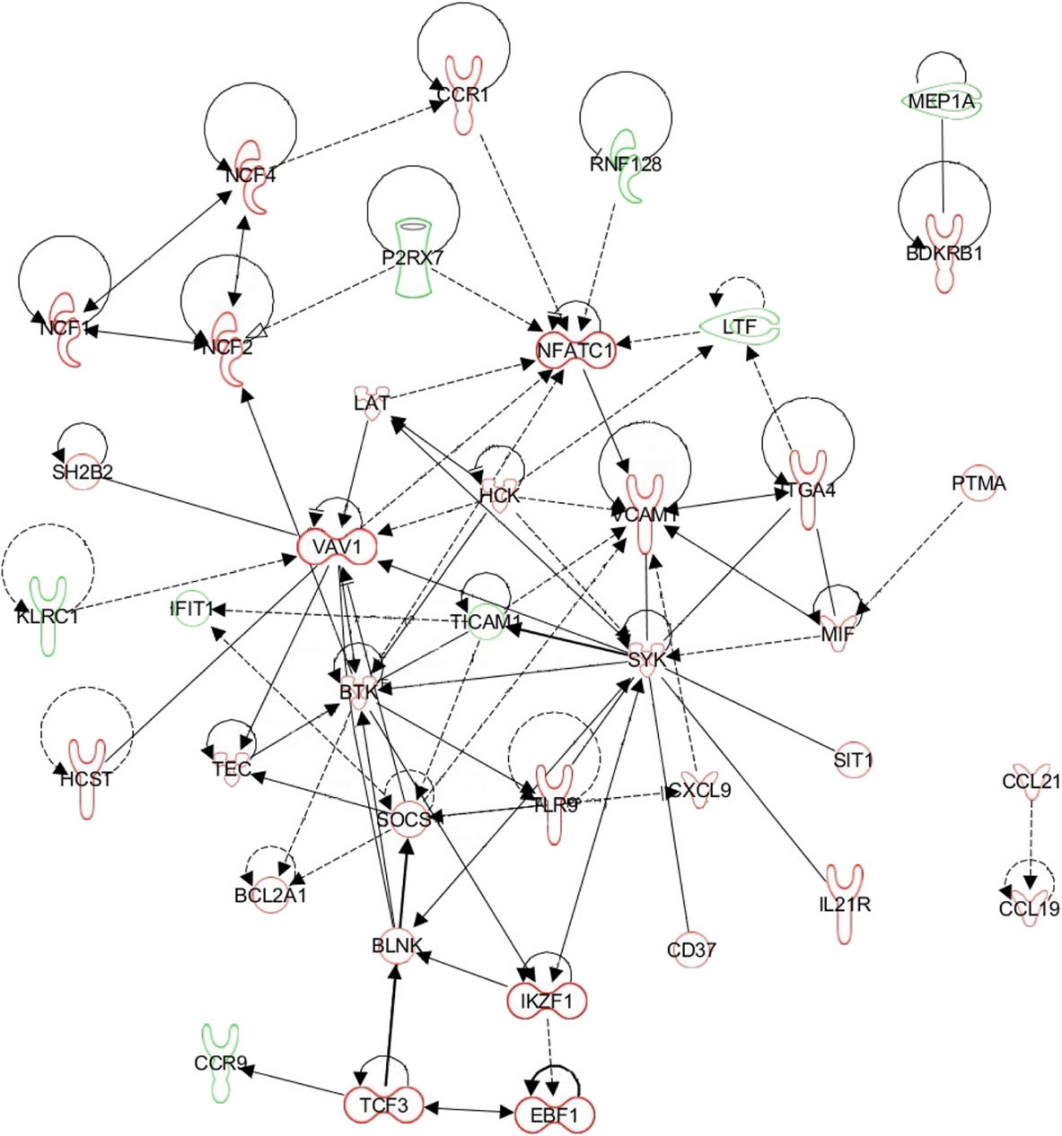
Regulatory network related to inflammatory response impacted in the ileal Peyer’s patches of calves after a primary infection with *E. coli* O157:H7. Up- and downregulation is represented by green and red colours, respectively

**Fig. 7:**
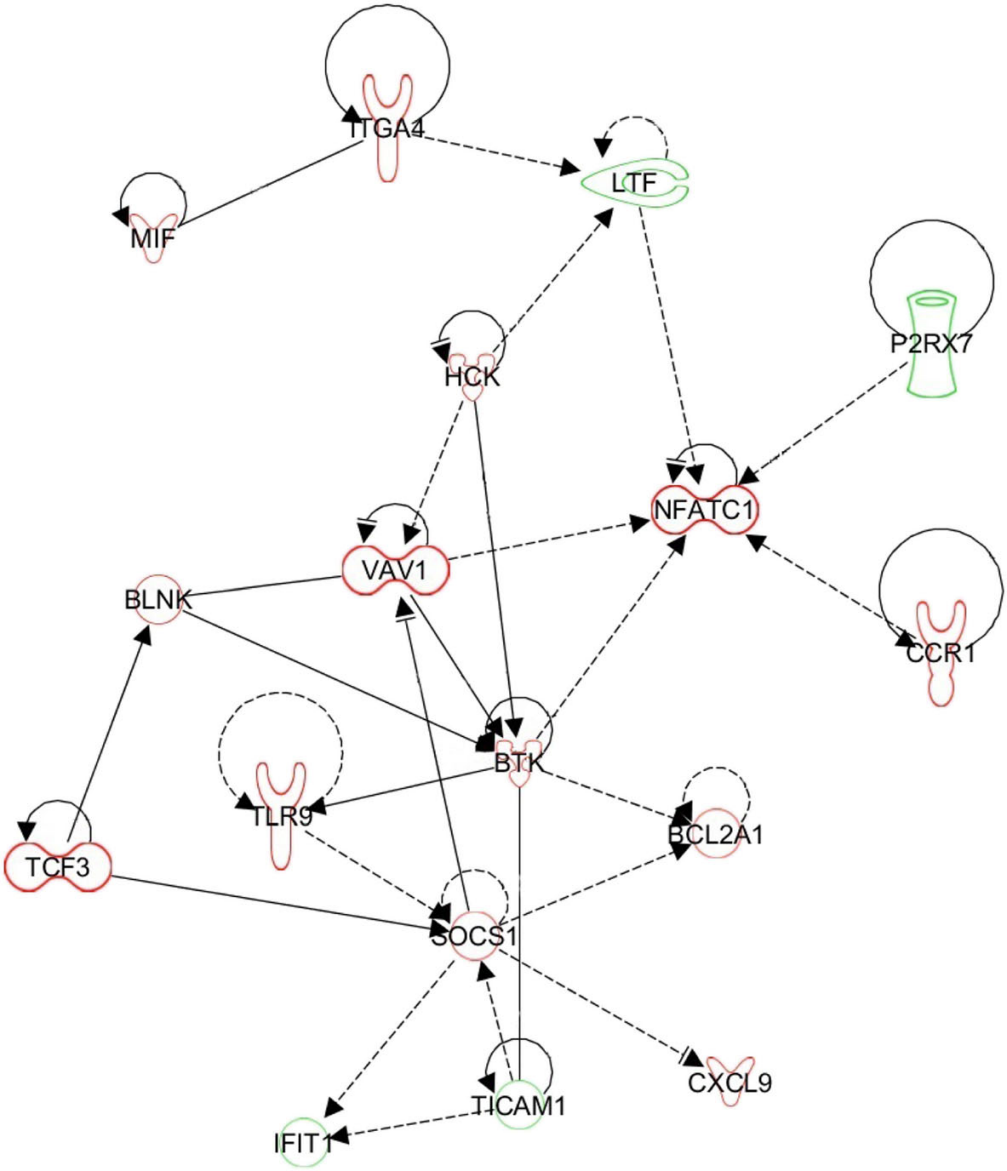
Regulatory network related to infectious disease impacted in the ileal Peyer’s patches of calves after a primary infection with *E. coli* O157:H7. Up- and downregulation is represented by green and red colours, respectively

The IPA software was used to further examine the RNA-Seq dataset. Uploading the dataset for the RAJ after a primary infection, the database could assign two relationships between the significantly regulated genes, whereas for the re-infection four relationships could be detected in both cases linked to antimicrobial responses. When the data for ileal Peyer’s patches was uploaded, IPA could identify three different networks that might play a role in EHEC infection, related to antimicrobial response (28 relationships) (Fig. 5), inflammatory response (69 relationships) (Fig. 6) and infectious disease (166 relationships) (Fig. 7).

### Possible effect of *E. coli* O157:H7 experimental infection on the function of immune cells

In order to gain insight in the impact of the differential mRNA expression, the data were arranged according to their effect on different immune cells in the specific tissues: lymphocytes (3.5.1), natural killer cells (3.5.2), monocytes and macrophages (3.5.3), dendritic cells (3.5.4), granulocytes (3.5.5) Only genes with the highest fold changes per cell type are reported. The gene expression results for RAJ and ileal Peyer’s patches after primary infection are represented in Tables 3 and 4, respectively.

**Table 3:** Gene expression results for recto-anal junction samples with fold change >2

Gene name^a^	Gene function	Fold change	Reference
KLRJ1 + (Ly49)	Natural killer cell receptor binding host MHC I as a mechanism of self/health recognition. Binding of the ligand results in an inhibitory signal to prevent killing of the target cell.	−3.89971	Storset et al. (2003) [27]
MARCO^−^	Innate immune defense. Can bind Gram-negative bacteria to stimulate clearing of the pathogen.	3.68421	Elomaa et al. (1998) [32]
CCL20^−^	Strongly chemotactic for lymphocytes and weakly for neutrophils.	3.33712	Mohammed et al. (2007) [22]

^a^: ^+^ = upregulated; ^−^ = downregulated

**Table 4:** Gene expression results for Ileal Peyer’s patches samples with fold change >2

Gene Name^a^	Gene function	Fold change	Reference
ENPP7^+^	Might have an inflammatory effect, as it could degrade and inactivate platelet-activating factor (PAF).	−9.92813	Wu et al. (2006) [55]
PDIA2^+^	Helps to load antigenic peptides into MHC I molecules and is therefore important in antigen recognition and clearing.	−4.4116	LeBrasseur (2006) [56]
BT.36112^+^	(KIR) Killer cell immunoglobulin receptor suppresses the cytotoxic activity of NK cells.	−2.92833	Vilches et al. (2002) [57]
MGC137099^+^	Is preferentially expressed on Th2 cells and is together with SEMA4A a stimulatory molecule for T-cell activation.	−2.54481	European bioinformatics institute (2015) [58]
PRLR^+^	Cytokine receptor and important in the JAK-STAT, JAK-RUSH, Ras-Raf-MAPK and PI-3 K pathways.	−2.47279	Bouchard et al. (1999) [59], Lee et al. (1999) [60], Amaral et al. (2004) [61]
LY6G6E^+^	Possible role of Ly-6 family members in T-cell activation, differentiation and maturation (mouse studies)	−2.4165	Mallya et al. (2006) [62]
BTRAPPIN-5^+^	Multifunctional host-defense peptide with anti-proteolytic, anti-inflammatory and anti-microbial activities.	−2.25014	Kato et al. (2010) [63]
SUSD2^+^	Contributes to evasion of immune responses by induction of apoptosis in activated T-cells	−2.24943	Watson. (2011) [23]
KLRJ1^+^	(see Table 3 on RAJ)	−2.04608	Storset et al. (2003) [27]
FCRLA^−^	Leading to inflammatory responses and antibody-mediated cellular cytotoxicity.	7.21319	Inozume et al. (2007) [64]
CXCL13^−^	Chemokine B-lymphocyte chemoattractant.	6.63854	Legler et al. (1998) [65]
DEFB5^−^	Bovine neutrophil β-defensins exert broad spectrum of antimicrobial activities against several species that cause mastitis as *S. aureus, E. coli, K. pneumoniae* and *P. aeruginosa*	6.0118	Alnakip et al. (2014) [66]
BT.53744^−^	Development and differentiation of B-cells into plasma cells.	6.01462	International Molecular Exchange Consortium (2015) [67]
TNFRSF13C^−^	Mature B-cell survival.	5.9961	Thompson et al. (2001) [68]
CD79B^−^	Initiation of the signal transduction cascade activated by the B-cell antigen receptor complex which will lead to antigen presentation.	5.81055	Luisiri et al. (1996) [69], Tseng et al. (1997) [70], Pelanda et al. (2002) [71]
SRCRB4D^−^	Regulation of innate and adaptive immune responses.	5.45381	OMIM database (2004) [72]
CD180^−^	Controls B-cell recognition and signaling of LPS.	4.59918	NCBI Reference Sequence Database (2008) [73]
FCRL1^−^	Functions in B-cell activation and differentiation.	4.43127	Gauld et al. (2002) [74], Harwoord et al. (2010) [75]
CLEC4E^−^	Induces secretion of inflammatory cytokines after binding of ligands (such as damaged cells, funghi and microbacteria).	4.4039	Miyake et al. (2010) [76]
CXCR5^−^	Chemokine plays an essential role in B-cell migration.	4.00308	Sáez de Guinoa et al. (2011) [25]
CD19^−^	Acts as a B-cell coreceptor in conjunction with CD21 and CD81.	3.8374	Van Zelm et al. (2006) [77]
P2RY8^−^	Regulator of the immune response.	4.05228	Amisten et al. (2007) [78]
LTA^−^	Mediates a large variety of inflammatory, immunostimulatory and antiviral responses.	3.70982	NCBI Reference Sequence Database (2012) [79]
CXCR4^−^	Receptor for SDF-1, has potent chemotactic activity for lymphocytes.	3.67364	Tamamis et al. (2014) [80]
TLR10^−^	Role in pathogen recognition and activation of innate immunity.	3.64532	Lee et al. (2014) [81]
SPP1^−^	Chemotactic for many cell types including macrophages, dendritic cells and T cells; it enhances B lymphocyte immunoglobulin production and proliferation. In inflammatory situations it stimulates both pro- and anti-inflammatory processes.	3.49813	Wang et al. (2008) [82]
BANK1^−^	Is expressed during development of B-lineage cells.	3.34384	Dymecki et al. (1992) [83]
FCRL3^−^	Regulator of the immune system.	3.32282	Swainson et al. (2010) [84]
LTB^−^	LTs are important for innate and adaptive immune responses by controlling the expression of several adhesion molecules, other cytokines and chemokines	2.50217	Creus et al. (2012) [85]
DOK3^−^	Negative regulator of JNK signaling in B-cells.	3.02771	Robson et al. (2004) [86]
KLRF1^−^	Activating lectin-like receptor expressed on NK-cells and stimulates their cytotoxicity and cytokine release.	3.00125	Kuttruff et al. (2009) [31]
FCER2^−^	Transportation in antibody feedback regulation	2.85172	Kijimoto-Ochiai (2002) [87]
FCAMR^−^	is expressed constitutively on the majority of B-lymphocytes and macrophages; FCAMR functions as a receptor for the Fc fragment of IgA and IgM and binds IgA and IgM with high affinity and mediates their endocytosis	2.82211	Shibuya et al. (2000) [88], McDonald et al. (2002) [89]
CCL19^−^	Antimicrobial gene; may play a role in normal lymphocyte recirculation and homing. It also plays an important role in trafficking of T cells in thymus, and in T cell and B cell migration to secondary lymphoid organs	2.54095	National Center for Biotechnology Information Gene (2014) [90]
TNFSF8^−^	Involved in cell differentiation, apoptosis and immune response	2.43376	Wei et al. (2011) [91]
SOCS1^−^	Negative regulator of cytokine signaling.	2.33913	Krebs et al. (2011) [92]
TIMD4^−^	Enhances the engulfment of apoptotic cells: involved in regulating T-cell proliferation and lymphotoxin signaling.	2.304	Uniprot (2015) [93]
SIT1^−^	Negatively regulates T-cell receptor mediated signaling in T-cells.	2.28439	Marie-Cardine et al. (1999) [94]
BDKRB1^−^	Receptor binding leads to increase in the cytosolic calcium ion concentration, resulting in chronic and acute inflammatory responses.	2.26204	Talbot et al. (2012) [95], Enquist et al. (2014) [96]
AKAP5^−^	Is expressed in T-lymphocytes and may function to inhibit IL-2; IL-2 is part of the body’s natural responses to microbial infections.	2.20946	Schillace et al. (2002) [97]
PGLYRP2^−^	Recognizes peptidoglycan, a component of bacterial cell walls.	2.19916	Dziarski et al. (2010) [98]
CD37^−^	T-cell and B-cell interactions.	2.13192	Knobeloch et al. (2000) [99]

^a^: ^+^ = upregulated; ^−^ = downregulated

#### Lymphocytes

In the RAJ, a significant downregulation of *Chemokine C-C motif ligand 20 (CCL20)* (fold change = 0.07) was observed, which is strongly chemotactic for immature dendritic cells, and B- and T-lymphocytes [22].

Furthermore, in the ileal Peyer’s patches, *Sushi domain containing 2 (SUSD2)* was significantly upregulated (fold change = 4.75). *SUSD2* can interact with *Galectin-1* which is known to contribute to the evasion of immune responses of tumors and infectious organisms by inducing apoptosis of activated T cells [23]. The activation of T-lymphocytes might be inhibited by the significant downregulation of the expression of *T cell immunoglobulin and mucin domain 4 (TIMD4)* (fold change = 0.20), responsible for regulation of Th1 responses [24]. *C-X-C motif ligand 13 chemokine (CXCL13)*, strongly expressed in the follicles of the spleen, lymph nodes and Peyer’s patches promoting the migration of B lymphocytes in the lymph nodes [25] was significantly downregulated in the ileum (fold change = 0.01). *Interleukin 17 receptor E-like (IL17REL)*, a member of the *Interleukin 17 (IL17)* cytokine receptor family that functions as a receptor for the proinflammatory cytokine responding to invading extracellular pathogens [26] was found to be significantly downregulated (fold change = 0.29) upon *E. coli* O157:H7 infection. A significant downregulation of *prothymosin alpha (PTMA)*, a tumor necrosis factor receptor (*RELT*), *Interleukin-21 receptor (IL21R)*, a guanine nucleotide exchange factor (*VAV1*) with fold change = 0.41, 0.38, 0.36, 0.35 respectively and many more immune response stimulating genes linked with lymphocyte responses were seen in the ileal Peyer’s patches.

#### Natural Killer (NK) cells

Effector functions of NK cells are controlled by a balance of inhibitory and stimulatory signals. In the RAJ, a strong significant upregulation of *killer cell lectin-like receptor (KLRJ1)* was observed (fold change = 14.93). *KLRJ1* is probably important for the NK cell recognition of target cells, which are certain tumor cells, virally infected cells and host MHC class I cells as a mechanism of self/health recognition. An upregulation of *KLRJ1* would imply an upregulation of the inhibitory signal, causing more survival of the target cells [27]. In the ileal Peyer’s patches, a significant upregulation of *protein disulfide isomerase (PDIA2)* (fold change = 21.28), *killer cell immunoglobulin-like receptor, two domains, short cytoplasmic tail 1 (KIR2DS1)* (fold change = 7.61), *killer cell immunoglobulin-like receptor, two domains, long cytoplasmic tail 5A (KIR2DL5A)* (fold change = 3.52) and *killer cell lectin-like receptor subfamily C, member 1-like (KLRC1) (fold change = 2.58)* was observed. In contrast, a significant downregulation of *IL21R* (fold change = 0.36) important for the proliferation and differentiation of B-, T- and NK cells, *leukocyte-associated immunoglobulin-like receptor 1 (LAIR1)* (fold change = 0.29) an inhibitory receptor found on NK cells, T cells and B cells regulating the immune response to prevent lysis of cells recognized as self [28], *hematopoietic cell signal transducer (HCST)* (fold change = 0.24) playing a role in cell survival and proliferation by activation of NK and T cell responses [29, 30] and *killer cell lectin-like receptor subfamily F, member 1 (KLRF1)* (fold change = 0.12) stimulating the cytotoxicity and cytokine release of NK cells [31], was observed.

#### Monocytes and macrophages

In the RAJ, *macrophage receptor with collagenous structure (MARCO)* was significantly downregulated (fold change = 9.20). This is a receptor which is part of the innate antimicrobial immune system binding both Gram-negative and Gram-positive bacteria via an extracellular, C-terminal, scavenger receptor cysteine-rich (*SRCR*) domain [32].

At the ileal Peyer’s patches significant upregulation was seen of *ectonucleotide pyrophosphatase/phosphodiesterase 7 (ENPP7)*, *PDIA2*, *carboxypeptidase M (CPM)* important for monocyte to macrophage differentiation [33], the inflammatory *purinergic receptor ligand-gated ion channel, 7 (P2RX7)* and *egf-like module containing, mucin-like, hormone receptor-like 4 pseudogene (EMR4P)*, a member of the *EGF-TM7* receptor gene family which is thought to be important for adhesion and migration of macrophages [34] with fold changes of 974.24, 21.28, 3.33, 3.24 and 2.64, respectively. Al these genes result in stimulation of the immune system on level of monocytes and macrophages. On the other hand, *macrophage migration inhibitory factor (MIF)* which is important for the acute immune response [35] was significantly downregulated as well as *chemokine C-C motif receptor 1 (CCR1)* playing a role in recruitment of leukocytes to the effector site [36] (fold change = 0.45 and 0.36, respectively). Furthermore, *sialic acid binding Ig-like lectin 10 (SIGLEC10)* which is a negative regulator of immune signaling by functioning as an inhibitory receptor [37] and *B cell linker (BLNK)*, an adaptor molecule linked to the pathway activated by B-cell antigen receptor signals [38], were significantly downregulated, with fold changes of 0.01 and 0.26, respectively.

#### Dendritic cells (DC’s)

No differential mRNA expression of genes indicating an effect on DC’s was seen in the RAJ. In samples from the ileal Peyer’s patches, a significant upregulation of *PDIA2*, *toll-like receptor adaptor molecule 1 (TICAM1)* (fold change = 2.97) and *EMR4P* was observed, whereas *SIGLEC10* and *MIF* were significantly downregulated.

#### Granulocytes

In the RAJ, there was no differential regulation observed that could have a direct influence on granulocytes. However, in the ileal Peyer’s patches, a significant upregulation of *ENPP7*, *PDIA2*, *chemokine C-C motif ligand 24 (CCL24)* (fold change = 3.82), which is chemotactic for eosinophils and neutrophils [39] and of *EMR4P* was detected. While a significant downregulation of *neutrophil cytosolic factor 1 (NCF1)* (fold change = 0.31), *neutrophil cytosolic factor 2 (NCF2)* (fold change = 0.31) and *neutrophil cytosolic factor 4 (NCF4)* (fold change = 0.43) important for the formation of the neutrophil phagosome leading to phagocytosis of bacteria [40], *CCR1*, *hemopoietic cell kinase (HCK)* (fold change = 0.31) playing a role in the neutrophil migration and degranulation [41], *SIGLEC10* and *vascular cell adhesion molecule 1 (VCAM1)* (fold change = 0.88) mediating the adhesion of lymphocytes, monocytes, eosinophils and basophils to vascular endothelium [42], was noticed.

## Discussion

It is well known that some pathogens have developed mechanisms to prolong their persistence in a host and can modulate the host immune response in different ways in order to increase their survival in the host [43]. This might be done by passive evasion of the immune surveillance for instance by altering expressed antigens, as *Streptococcus pneumonia* does. Another way to survive is actively modulating and interfering with regulatore networks that are part of the immune defence. This is done when EHEC and EPEC inject modulatory proteins into the host cell using T3SS [43–45]. Furthermore, the suppression of immune responses via TLR4 by uropathogenic *E. coli* (UPEC) leads to a decrease of IL-6 and IL-8 release [46]. This is congruent with our observations in several infection experiments: a primary infection could elicit an antibody response, but this primary immune response was unable to protect against an experimental re-infection with the same strain. On the contrary, whereas the primary infection led to faecal shedding during less than 2 weeks (<14 days; *n* = 32), a re-infection resulted in excretion for more than 4 weeks (>28 days; *n* = 21) (Table 5; unpublished results).

**Table 5:** Duration of infection after primary or re-infection with NCTC12900 strain

Duration of infection	Primary infection (*n* = 32)	Re-infection (*n* = 26)
0–7 days	9	0
7–14 days	23	5
14–21 days	0	4
21–28 days	0	3
>28 days	0	14

To our knowledge, this is the first RNA-Seq study highlighting the effect of a primary infection and a re-infection with *E. coli* O157:H7 in cattle, leading to a better understanding of the transient and sometimes recurrent pattern of EHEC infections. In 2007, Li and Hovde described the differential expression of 49 genes in the RAJ after a primary infection with *E. coli* O157:H7 using cDNA microarray [47]. Here, we used the RNA-Seq technique which allowed us to determine effects on both RAJ and ileal Peyer’s patches. We could identify an important difference in regulation of the transcriptome after an initial contact with the bacteria: the ileal Peyer’s patches were more influenced by the infection compared to the RAJ, which is part of the primary site for colonization in cattle [10]. In this study we were able to show suppression of immunity on different levels of the innate and adaptive immune response, indicating that *E. coli* O157:H7 can modulate this response in a wide variety of ways. The excretion patterns confirm our previous findings, as a first infection mounts a higher level of bacterial shedding during a shorter period of time compared to a re-infection. It might be mooted that there is a correlation between the higher level of infection and a more pronounced effect on the immune system. However, we hypothesize that the priming of the immune system by the first exposure is more important than the level of bacteria since a second infection with *E. coli* O157:H7 mostly results in a prolongued colonization, whereas for other pathogenic *E. coli* such as enterotoxigenic *E. coli* in pigs a complete protection occurs after a first infection.

Our data suggests that an infection with *E. coli* O157:H7 (Stx^−^) is capable of modulating the immune response causing dramatic decreases in *CCL20*. A similar trend was observed in a bacterial infection with *Mycoplasma gallisepticum* in chicken, where a down-regulation in mRNA expression of *CCL20* as well as of *IL-1*, *IL-8* and *IL-12p40* genes was seen. These results indicate the importance of lymphocyte and monocyte chemotactic factors in development of disease, but also the fast occurrence of modulations of the host immune responses by bacteria [22].

Granulysin delivers granzymes into bacteria to kill diverse bacterial strains. In *Escherichia coli*, granzymes cleave electron transport chain complex I and oxidative stress defense proteins, generating reactive oxygen species (ROS) that rapidly kill bacteria [48]. An upregulation of granulysin is part of the adaptive immune response against bacterial infections.

A study of St John et al. [49] demonstrated downregulation of *CXCL13* and *CCL21* during an infection of draining lymph nodes by *Salmonella typhimurium*. The pathogen disrupts the lymph node architecture and cellular trafficking, which enhances its virulence and could serve as a mechanism of immune suppression used by pathogens that primarily target lymphoid tissue. In our study, we could not observe a significant downregulation of *CXCL13* nor *CCL21* altough the bacterium is in close contact to lymphoid dense tissue in the RAJ and the ileum.

We observed a downregulation of *IL17REL*, a gene which is important for a fast inflammatory response. The cells of the innate immune system are the first line of defense against pathogen and their cytokines govern the differentiation of T- helper cells. Their pattern-recognition receptors, which are not specific for any particular epitope, allow them to respond to a wide variety of microbial invaders by producing cytokines that activate T-cells of the adaptive immune system. T-helper 17 cells produce IL17 and this is particularly important for immunity at epithelial and mucosal surfaces, as indicated by the pattern of expression of their chemokine receptors and effector cytokines. Several pathogens, like gram-positive *Propionibacterium acnes* and gram-negative *Citrobacter rodentium*, induce mainly Th17 responses [50]. Furthermore, Luo et al. [51] have shown that F4 fimbriae of ETEC can elicit an IL17 response in piglets, suggesting a role in protection of the host against ETEC infection.

A downregulation of *IL21R* might play a role in the persistence of colonization during the primary *E. coli* O157:H7-infection and re-infection of the host, since the IL-21-IL-21R pathway is important in the development of immune responses, as abnormal signaling through the IL-21R/γc/JAK3/STAT3 pathway leads to defective humoral immune responses to both T-dependent and T-independent antigens and impairs the establishment of long-lasting B-cell memory [52]. A bacterial infection can elicit IgM memory B-cells which requires T cell- dependent and IL-21R signaling. The study of Yates et al. [53] demonstrates that T cell-dependent IgM memory B cells can be elicited at high frequency and can play an important role in maintaining long-term immunity during bacterial infection.

In the RAJ, we could observe the downregulation of *MARCO*, a receptor that can bind Gram-negative bacteria and is only found on macrophages of the marginal zone of the spleen and lymph nodes [32]. Pinheiro da Silva et al. [54] have shown that *E. coli* are capable of hijacking inhibitory ITAMs leading to a decrease in MARCO-mediated phagocytosis. This observation is indicating a decrease in antibacterial protection of the host at the primary site of EHEC infection.

Due to biosafety reasons and limitations on housing facilities we performed this experiment using an *E. coli* O157:H7 (Stx^−^) strain. Although this is not the exact same strain as the strains found during natural EHEC-infections, this study may be seen as a first step to investigate the immunomodulating capacities of *E. coli* O157:H7 strains in cattle using RNA-Seq. We believe that this study is a valuable contribution to the current knowledge on immune suppression in the bovine host and this study is congruent with previous findings showing that also other bacterial factors apart from Stx can play a role in immune suppression.

A limitation of our study is that most information from gene function databases is derived from studies that are not performed in cattle but in human and mice, therefore we have to take into consideration species-specific differences which we unfortunately can not control. This study is important since it is the first study using the highly-accurate and sensitive RNA-Seq technique to study the effect of EHEC on the cattle immune responses. These insights are crucial to better understand EHEC colonization and shedding within herds and our data could contribute to effective measures to control EHEC colonization in ruminants, thereby reducing zoonotic food-borne infections in humans.

## Conclusions

We can conclude that the main effect on the transcriptome was immune suppression by *E. coli* O157:H7 due to an upregulation of immune suppressive effects (7/12 genes) or a downregulation of immunostimulatory effects (69/94 genes). Furthermore, the changes in gene expression were remarkably higher in the ileal Peyer’s patches (1159 genes) than in the RAJ (15 genes) during a primary infection. This effect was less obvious after the re-infection (17 and 10 genes, respectively). The data might indicate that a primary infection promotes a re-infection with EHEC by suppressing the immune function.

## Abbreviations

CT-SMAC: Cefiximin Tellurite - Sorbitol MacConkey agar
DC’s: Denditic cells
*E. coli*: *Escherichia coli*
EHEC: Enterohaemorrhagic *Escherichia coli*
EPEC: Enteropathogenic *Escherichia coli*
Esp: *E. coli* secreted protein
FDR: False discovery rate
GO: Gene ontology
IMS: Immunomagnetic separation
IPA: Ingenuity pathway analysis
LB: Luria Bertani broth
LEE: Locus of enterocyte effacement
LPS: Lipopolysaccharide
MHC: Major histocompatibility complex
mRNA: Messenger RNA
NK-cells: Natural killer cells
PBS: Phosphate buffered saline
RAJ: Recto-anal junction
RNA-Seq: RNA-sequencing
ROS: Reactive oxygen species
Stx: Shiga toxin
T3SS: Type III secretion system
Th: T-helper
Tir: Translocated intimin receptor
TLR: Toll-like receptor
UPEC: Uropathogenic *Escherichia coli*
